# In Memoriam

**Published:** 1890-08

**Authors:** 


### In Memoriam.

Dr. John Stephan died of consumption at his residence in Cleveland, Ohio, after a lingering illness of three years at the age of 41 years. Dr. Stephan was born in Essenheim, Germany, and at the age of three years came with his parents to Cleveland where, after his school days were ended, he commenced his single handed struggle with life's problem. He was first apprenticed to the machinist trade for which he had a strong liking, but not liking his employers, soon obtained his release and returned to the public schools to study in the higher grades. At this time his neighbors and play fellows were the sons of Dr. W. H. Atkinson which no doubt turned his thoughts towards dentistry and induced him to make that his life work. After a short pupilage with Dr. H. H. Newton he attended his first course at the Ohio Dental College. On his return he found himself without means to pursue his studies further and then with a determination which knew no such word as fail began the practice of dentistry among his boyhood friends. In a few years, aided by a faithful wife, he had secured the means to complete his course and receive the degree of D. D. S.

Dr. Stephan was a faithful worker in the field of dentistry, always striving to elevate his chosen professsion and was a faithful attendant on all meetings of the Northern Ohio and Cleveland Dental Societies, of the former of which he was the retiring president at its last meeting, but the disease that finally laid him low had so far obtained the mastery that he was unable to be present. Almost his last writing was a short address as its retiring president. Dr. Stephan leaves a widow and five children in comfortable circumstances. Two of his sons are now students in dentistry.

